# Concurrent transcranial direct current stimulation and progressive resistance training in Parkinson’s disease: study protocol for a randomised controlled trial

**DOI:** 10.1186/s13063-016-1461-7

**Published:** 2016-07-19

**Authors:** Ashlee M. Hendy, Alex Tillman, Timo Rantalainen, Makii Muthalib, Liam Johnson, Dawson J. Kidgell, Daniel Wundersitz, Peter G. Enticott, Wei-Peng Teo

**Affiliations:** School of Exercise and Nutrition Sciences, Deakin University, Melbourne, Australia; Institute for Physical Activity and Nutrition (IPAN), Deakin University, Burwood, 3125 Melbourne, VIC Australia; EuroMov, University of Montpellier, Montpellier, France; Clinical Exercise Science Research Program, Institute of Sport, Exercise and Active Living, Victoria University, Melbourne, Australia; Stroke Division, The Florey Institute of Neuroscience and Mental Health, The University of Melbourne, Melbourne, Australia; La Trobe Sport and Exercise Medicine Research Centre, School of Allied Health, La Trobe University, Melbourne, Australia; La Trobe Rural Health School, College of Science, Health and Engineering, La Trobe University, Bendigo, Australia; Cognitive Neuroscience Unit, School of Psychology, Deakin University, Melbourne, Australia

**Keywords:** Parkinson’s disease, Balance, Gait, Neuroplasticity, Non-invasive brain stimulation, fNIRS

## Abstract

**Background:**

Parkinson’s disease (PD) results from a loss of dopamine in the brain, leading to movement dysfunctions such as bradykinesia, postural instability, resting tremor and muscle rigidity. Furthermore, dopamine deficiency in PD has been shown to result in maladaptive plasticity of the primary motor cortex (M1). Progressive resistance training (PRT) is a popular intervention in PD that improves muscular strength and results in clinically significant improvements on the Unified Parkinson’s Disease Rating Scale (UPDRS). In separate studies, the application of anodal transcranial direct current stimulation (a-tDCS) to the M1 has been shown to improve motor function in PD; however, the combined use of tDCS and PRT has not been investigated.

**Methods/design:**

We propose a 6-week, double-blind randomised controlled trial combining M1 tDCS and PRT of the lower body in participants (*n* = 42) with moderate PD (Hoehn and Yahr scale score 2–4). Supervised lower body PRT combined with functional balance tasks will be performed three times per week with concurrent a-tDCS delivered at 2 mA for 20 minutes (a-tDCS group) or with sham tDCS (sham group). Control participants will receive standard care (control group). Outcome measures will include functional strength, gait speed and variability, balance, neurophysiological function at rest and during movement execution, and the UPDRS motor subscale, measured at baseline, 3 weeks (during), 6 weeks (post), and 9 weeks (retention). Ethical approval has been granted by the Deakin University Human Research Ethics Committee (project number 2015-014), and the trial has been registered with the Australian New Zealand Clinical Trials Registry (ACTRN12615001241527).

**Discussion:**

This will be the first randomised controlled trial to combine PRT and a-tDCS targeting balance and gait in people with PD. The study will elucidate the functional, clinical and neurophysiological outcomes of combined PRT and a-tDCS. It is hypothesised that combined PRT and a-tDCS will significantly improve lower limb strength, postural sway, gait speed and stride variability compared with PRT with sham tDCS. Further, we hypothesise that pre-frontal cortex activation during dual-task cognitive and gait/balance activities will be reduced, and that M1 excitability and inhibition will be augmented, following the combined PRT and a-tDCS intervention.

**Trial registration:**

Australian New Zealand Clinical Trials Registry ACTRN12615001241527. Registered on 12 November 2015.

## Background

Parkinson’s disease (PD) is a progressive neurodegenerative movement disorder characterised by depletion of dopamine in the substantia nigra, resulting in physical symptoms such as bradykinesia, postural instability, resting tremor and muscle rigidity. PD is the second most common neurodegenerative disorder [1], affecting 1 % of people older than 60 years of age [2], which is of particular concern as populations age. PD causes significant impairments in gait and balance, which are often unresolved by standard dopamine pharmacological treatment [3] and result in reduced quality of life and increased risk of falls [4].

There is growing evidence for the use of progressive resistance training (PRT) to improve gait and lower limb muscle strength in PD [5]. The inclusion of other functional tasks, such as balance training, in PRT programmes has also been shown to decrease postural sway, reduce the risk of falls, and improve quality of life [6]. Importantly, functional PRT programmes have been shown to induce neuroplastic changes in the primary motor cortex (M1) of both healthy individuals [7] and people with PD [8]. This is of particular interest, as maladaptive M1 plasticity in patients with PD has been linked to poorer outcomes in motor learning and movement control [9, 10]. Evidence derived from transcranial magnetic stimulation (TMS) studies has demonstrated that patients with PD present with increased cortical excitability and reduced inhibition at rest, which is evidenced by lower motor threshold, increased motor evoked potential (MEP) amplitude, reduced short-interval intra-cortical inhibition (SICI) [10] reduced silent period [11]. Furthermore, reduced intra-cortical facilitation and absence of MEP size increase during contraction suggests defective facilitatory cortical inputs, particularly for movement execution [12]. In addition, the absence of a putative neuroplastic response following paired associative stimulation [13, 14], as compared with healthy control subjects, suggests that the facilitation of M1 plasticity in patients with PD may play an important role in the restoration and maintenance of motor performance.

Transcranial direct current stimulation (tDCS) is a form of non-invasive brain stimulation that has been shown to modulate excitability of the M1 in a polarity-specific manner when applied for short periods (10–20 minutes). When anodal tDCS (a-tDCS) is applied to the M1, there is a transient increase (up to 90 minutes) in excitability and reduction SICI of underlying neurons [15], as well as widespread changes in activation of cortical and subcortical areas [16]. There is substantial evidence to suggest that the application of a-tDCS to the M1 results in improved motor function in healthy populations, including increased performance in skill and strength tasks [17, 18], and this may be especially true when tDCS is applied during concurrent motor practice or training [19].

The use of tDCS as an independent intervention in PD has produced promising results, with evidence for improved working memory and executive function [20, 21], reduced bradykinesia, and increased walking speed following tDCS [22]. Importantly, clinically significant improvements in the motor component of the Unified Parkinson’s Disease Rating Scale (UPDRS) have been reported following bilateral a-tDCS of the M1 [23]. A recent pilot study of eight participants reported for the first time that combined gait training and M1 a-tDCS showed promising improvements in walking and balance measurements, but the researchers in that study concluded that larger trials were required to produce definitive results [24].

Given the existing evidence for enhancements in neuroplasticity and motor performance following both PRT and tDCS as independent treatments, it is reasonable to conclude that the combination of these two interventions may further augment functional benefits in patients with PD. Indeed, a combination of a-tDCS and motor training appears to produce a compounding effect on motor performance and neurophysiological adaptations in healthy populations [25, 26] and stroke-affected individuals [27, 28]; however, the application of a-tDCS during PRT and balance training in PD is yet to be investigated.

## Methods/design

### Aims

The primary aim of this pilot study is to determine the effects of a 6-week lower limb balance and PRT intervention with concurrent a-tDCS on gait, balance, strength and UPDRS motor scores in patients with PD. In addition, we will investigate neurophysiological adaptations in M1 excitability and pre-frontal cortex (PFC) activation as potential underlying mechanisms. It is hypothesised that the combination of PRT and a-tDCS will produce favourable functional motor outcomes and neuroplasticity beyond the effects of PRT alone.

### Eligibility and recruitment

**Fig. 1:**
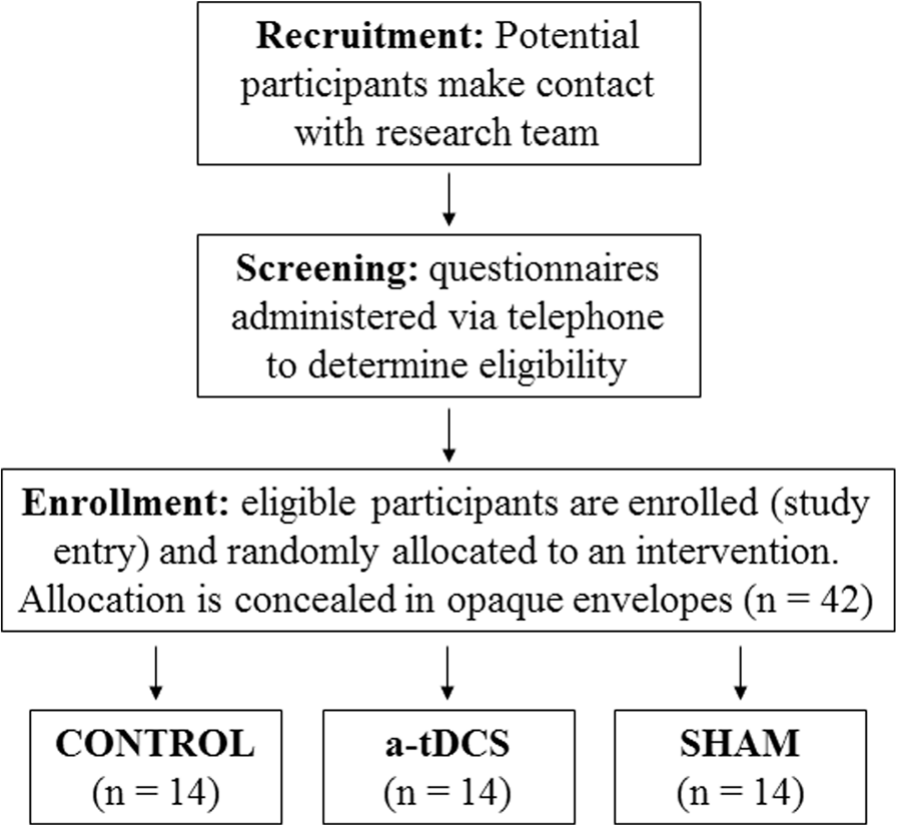
Flow diagram of recruitment, screening and randomisation process. *a-tDCS* anodal transcranial direct current stimulation

The recruitment, screening and randomisation process is shown in Fig. 1. Potential participants will be recruited on a voluntary basis with the assistance of the Australian Parkinson’s Disease Registry and neurology clinics within the Melbourne metropolitan area. All participants will provide written informed consent as well as written approval from their general practitioner before partaking in physical exercise. Prior to enrolment in the study, a series of screening questionnaires will be completed by telephone to determine eligibility.

The following inclusion criteria will be applied:

1. Diagnosed with PD by an independent neurologist
2. Moderate motor symptoms (score between 2 and 4 on the Hoehn and Yahr scale, as assessed by an independent neurologist and confirmed at initial assessment by a blinded researcher)
3. Stable drug regime for the full duration of the study and the 6 weeks preceding the study
4. Self-reported history of one or more falls in the last 24 months
5. Not currently undertaking a regular exercise programme

Potential participants will be excluded if they present with any of the following:

1. Severe motor impairments or injuries that will impair the ability to perform lower limb PRT (confirmed at initial assessment by blinded researcher)
2. Previously diagnosed neurological condition separate from PD (stroke, dementia, epilepsy)
3. Metal implants in the head (i.e., deep brain stimulator or aneurysm clips), which are contraindicated with non-invasive brain stimulation techniques

### Randomisation and blinding

Once recruited, simple randomisation using a computerised sequence generator will be used to allocate participants to one of three study groups (a-tDCS, sham or control). Opaque envelopes will be used to conceal the group allocation, which will not be revealed until after all data have been analysed. A researcher who is otherwise removed from the study will program the tDCS machine before PRT sessions to ensure double-blinding of both researcher and participant. Participants allocated to the control group will be notified of their allocation and will be asked to attend assessment sessions only.

### Study overview

The study will run for a period of 9 weeks in total, including a 6-week intervention period and a 3-week follow-up. Participants will be randomly allocated to one of three groups:

1. a-tDCS (required to perform PRT and balance training while receiving a-tDCS)
2. Sham (required to perform PRT and balance training while receiving sham tDCS)
3. Control (to receive standard care)

**Fig. 2:**
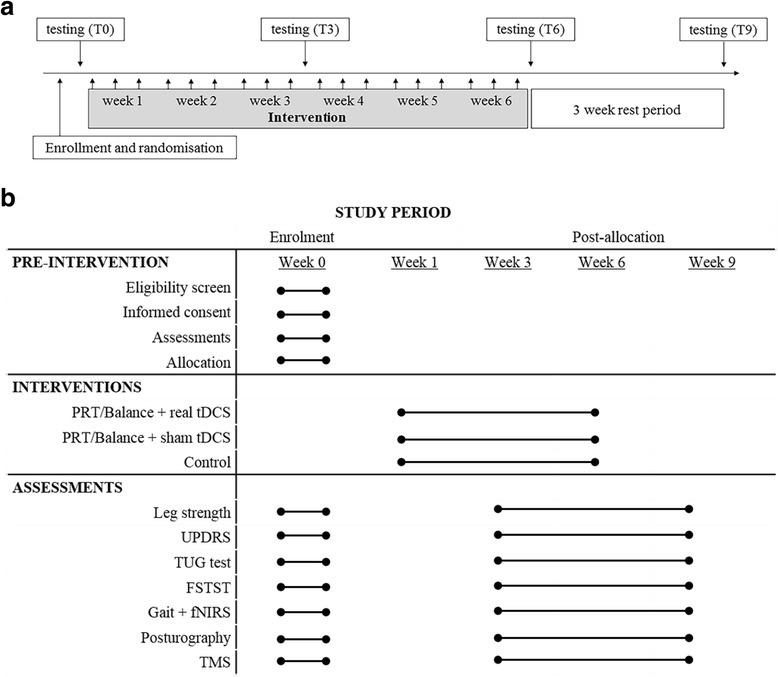
Study timeline (**a**) and Standard Protocol Items: Recommendations for Interventional Trials (SPIRIT 2013) diagram (**b**) illustrate the schedule of enrolment, interventions and assessments. *fNIRS* functional near-infrared spectroscopy, *FTSTS* Five Times Sit-to-Stand Test, *PRT* progressive resistance training, *tDCS* transcranial direct current stimulation, *TMS* transcranial magnetic stimulation, *TUG* Timed Up and Go Test, *UPDRS* Unified Parkinson’s Disease Rating Scale

Exercise sessions will include PRT and balance training for a duration of approximately 40 minutes, and they will be completed on 3 non-consecutive days per week (18 sessions in total). Assessment sessions to evaluate outcome measures will take place at four time points: baseline (T0), mid-intervention at 3 weeks (T3), post-intervention at 6 weeks (T6) and retention at 9 weeks (T9). Assessment sessions will take approximately 3 h and will occur at the same time of day for each time point. All sessions will take place at the Deakin University Burwood campus in a specialised clinical exercise gym and laboratory facility. The control group will receive standard care for the duration of the study and will be required only to attend the assessment sessions. Figure 2 provides an overview of the study time frame and sessions. For the duration of the study, all participants will continue with their usual care and medications as prescribed by their physician. Any changes in medication status during the trial will be documented, and participants will be allowed to continue with the programme.

### Intervention

#### Strength and balance training

**Fig. 3:**
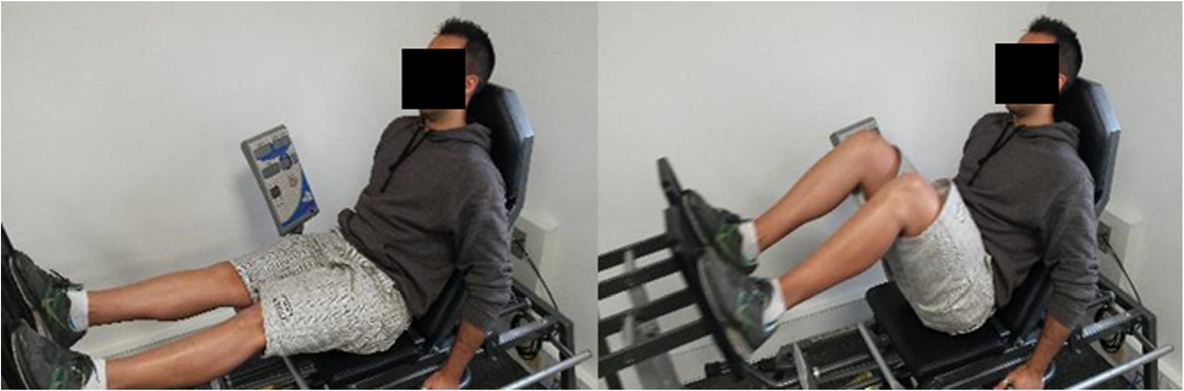
Example of seated leg press

**Fig. 4:**
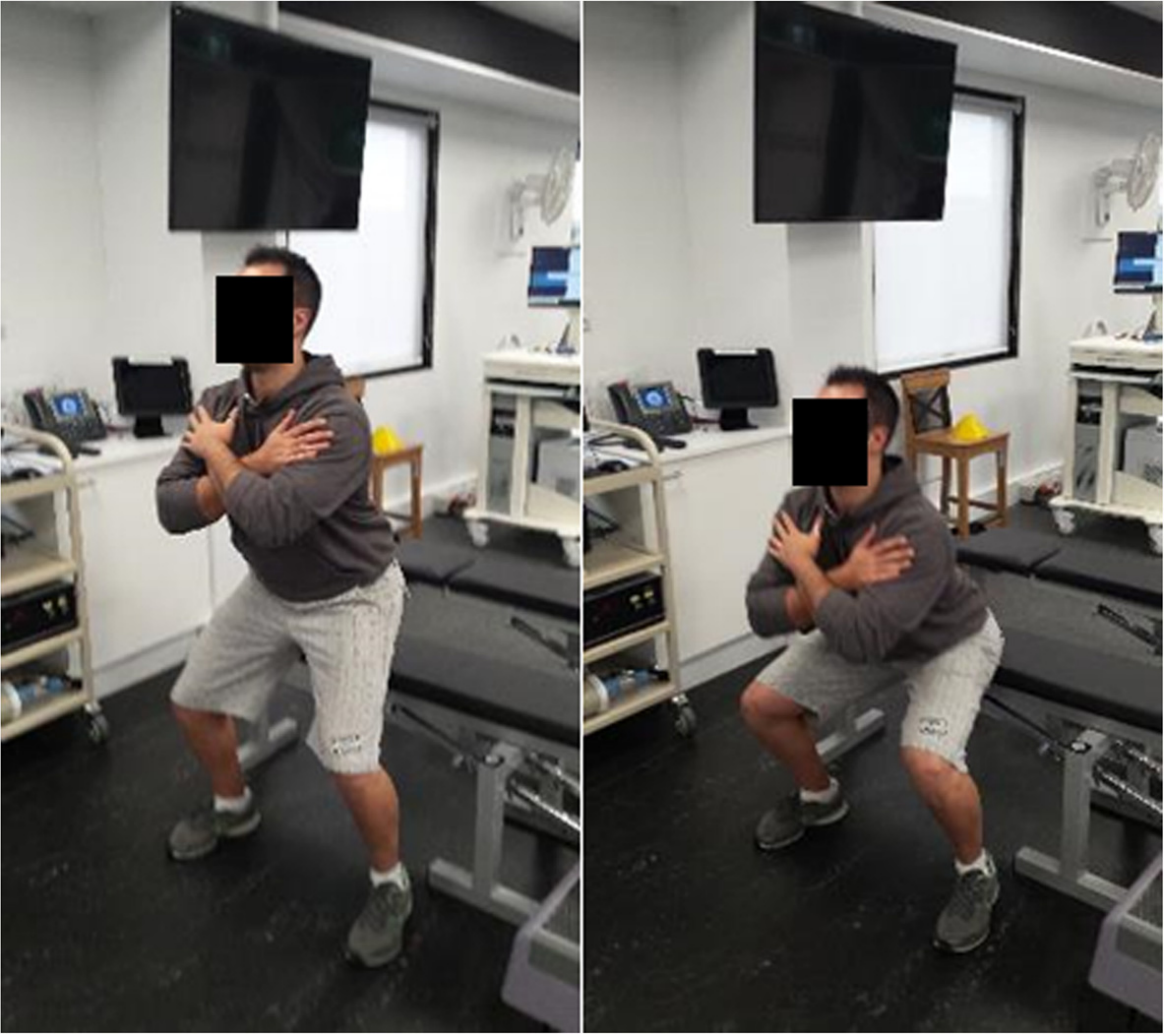
Example of body weight squat

**Fig. 5:**
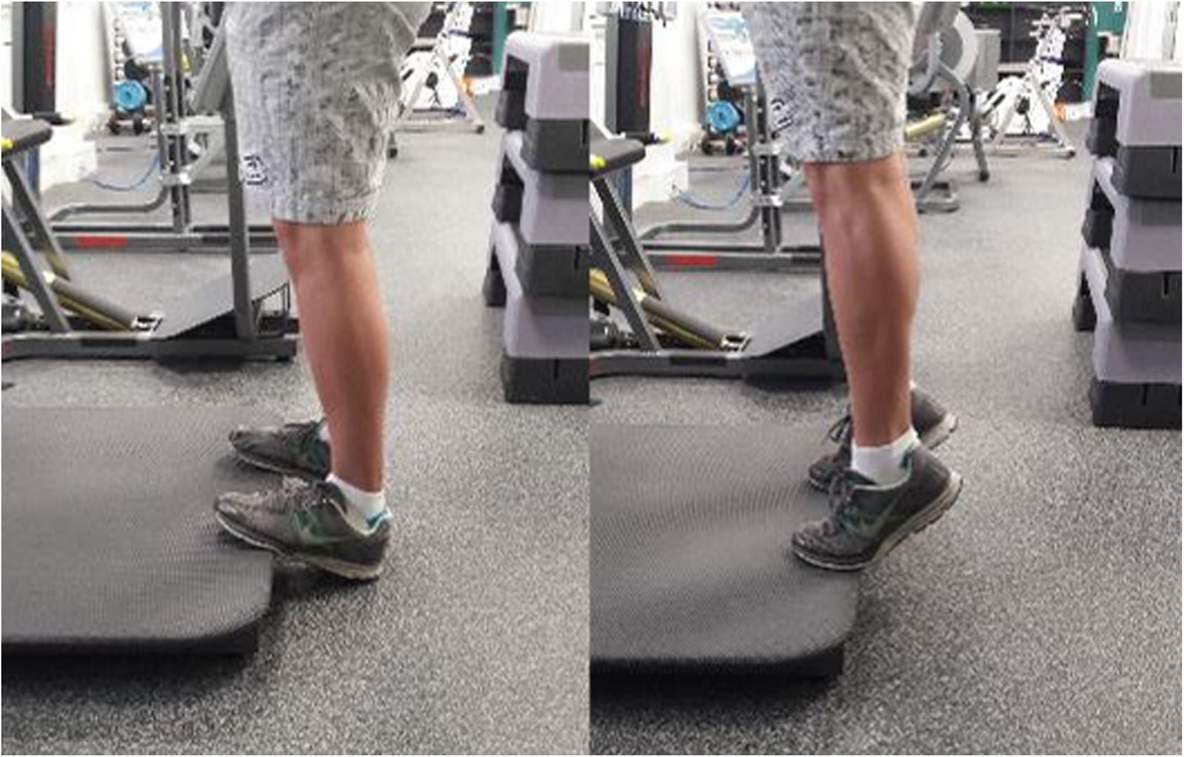
Example of standing bilateral calf raise

**Fig. 6:**
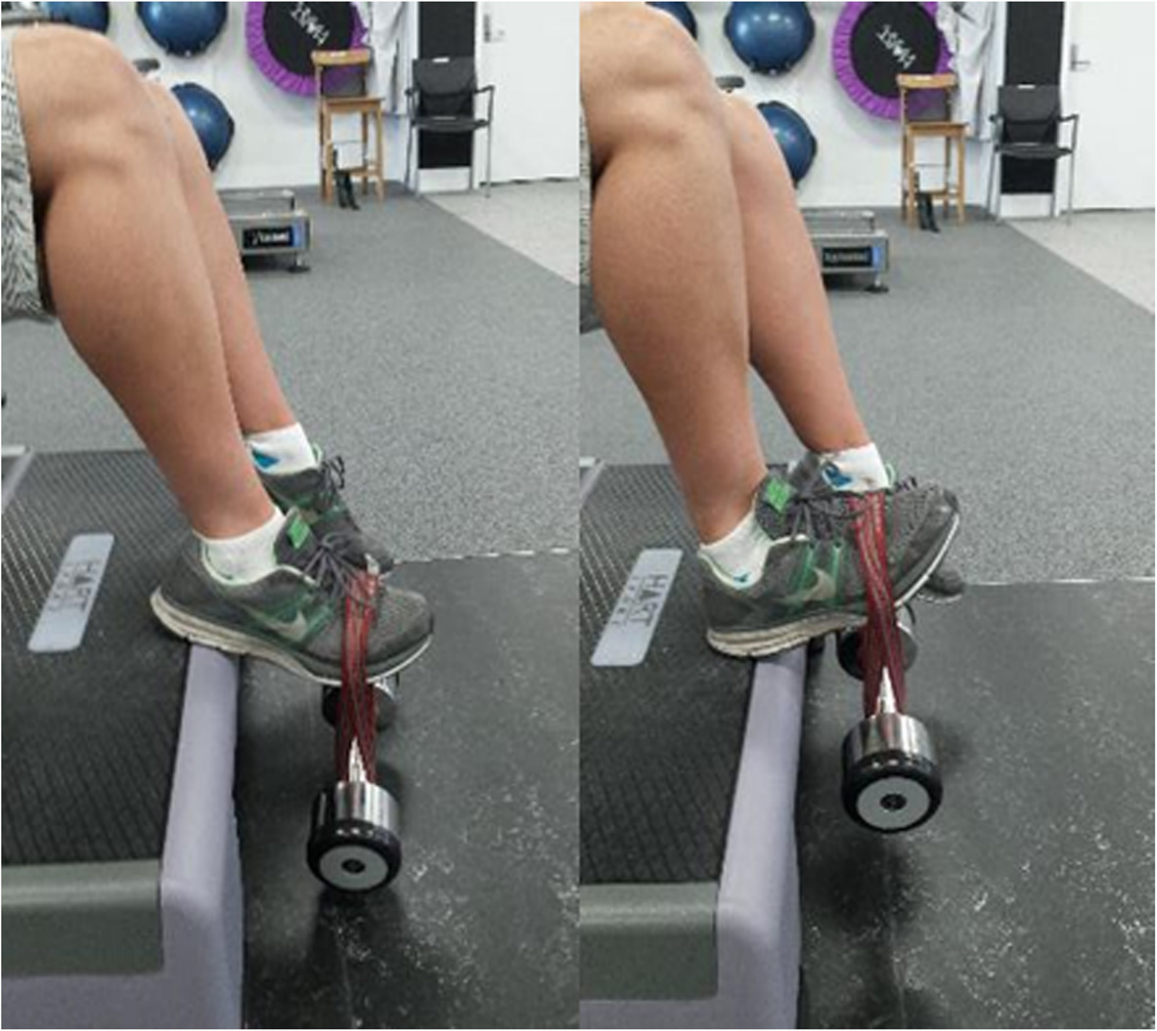
Example of unilateral dorsiflexion with free-weight dumb-bell

Participants will be required to attend PRT and balance training sessions at Deakin University Burwood campus three times per week, on non-consecutive days, for 6 weeks. All PRT exercises will be aimed at the lower body and will include the following: leg press performed on fixed pneumatic gym equipment (Air300; Keiser, Fresno, CA, USA) (see Fig. 3); sit to stand, progressing to body weight squat and addition of weight vest (5, 10 or 15 kg) (see Fig. 4); standing bilateral calf raise, progressing to unilateral calf raise and weighted vest (se Fig. 5); and seated unilateral dorsiflexion with free weight dumb-bell (see Fig. 6).

Participants will perform three sets of six to eight repetitions of each exercise, with resistance set at 70 % of their recorded single-repetition maximum (1RM). Repetition timing will be externally paced by an audible metronome consisting of 3-second concentric and 3-second eccentric contractions. For each exercise, resistance will be increased by 5 % when all sets can be completed with correct repetition timing and technique, as assessed by a trained exercise physiologist.

Between each set of exercises, participants will perform a functional balance task:

1. Tandem stance, 4 × 15 seconds alternating the leading foot
2. Single-leg stance, 4 × 15 seconds alternating the leading foot
3. Tandem walk, 3 × 12 steps, heel to toe, in a straight line
4. Hurdle walk, six laps of four hurdles (height = 30 cm)

Stance tasks will be performed with the assistance of the trainer if required and will be progressed by using unstable foam surfaces, duraDiscs (AOK Health, Newcastle, Australia), performing with the eyes closed, and receiving multi-directional perturbations. Walking tasks will be progressed by performing a concurrent dual task (naming colours, cities, countries, and so forth), adding 1-kg ankle weights, and being instructed to ‘pause’ and ‘reverse’ mid-task. Participants will advance to the next progression when the exercise physiologist deems that they can competently complete the task.

#### Transcranial direct current stimulation

**Fig. 7:**
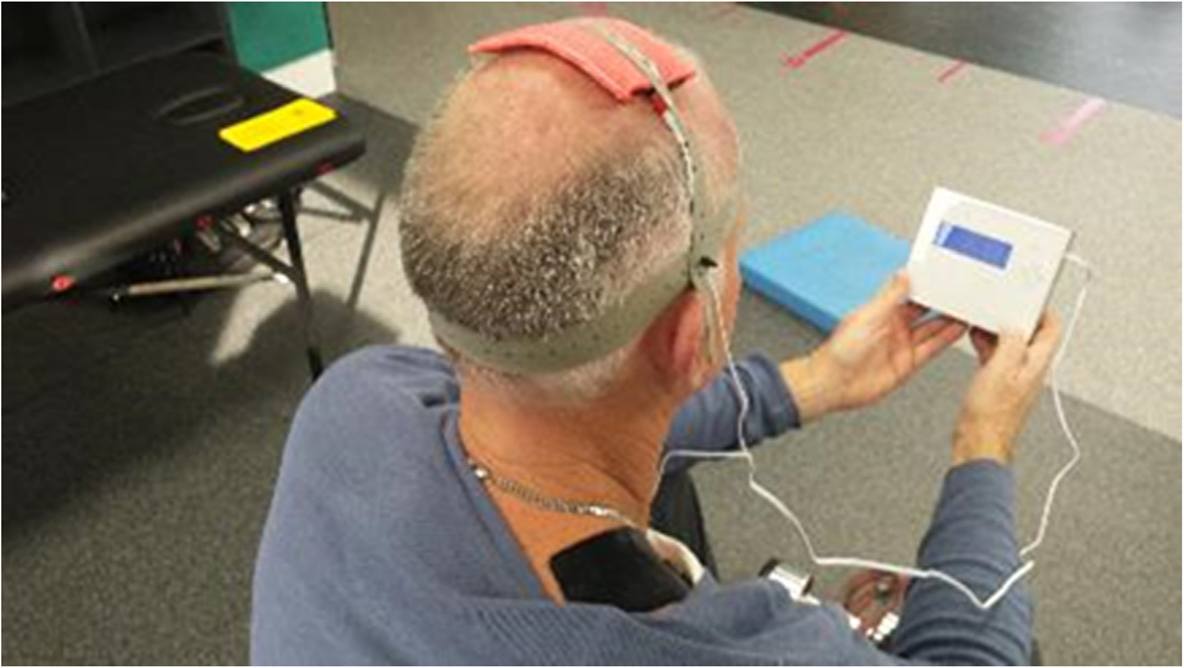
Example of electrode montage for anodal transcranial direct current stimulation

A tDCS Stimulator Model 101 (TCT Research Limited, Hong Kong, China) will be used to deliver a-tDCS and sham tDCS. For a-tDCS, a current of 2 mA will be delivered through two 50-cm^2^ electrodes, producing a current density of 0.04 mA/cm^2^. The anode will be soaked in saline and placed centrally over the left and right motor representation of the lower limb, as pre-determined with TMS, and secured with adjustable rubber straps. The cathode will be placed on the right trapezius muscle at the base of the neck, with Ten20 conductive gel (Weaver and Company, Aurora, CO, USA) used to secure the rubber electrode directly onto the skin. Figure 7 demonstrates the electrode montage. Stimulation will commence at the beginning of the training session and will be delivered for a duration of 20 minutes (typically ceasing approximately halfway through the session). Bioelectrical impedance will be monitored throughout the stimulation protocol and will not exceed 55 kΩ. Sham tDCS will involve an identical electrode montage, with stimulation ceasing after a 20-second ramp-up period to provide equivalent scalp sensation. A pseudo-stimulation feature built into the direct current stimulator allows for the device to be pre-programed to deliver sham stimulation while maintaining identical appearance, operation and screen prompts. This was conducted prior to each session by a laboratory assistant, enabling double-blinding of both researcher and participant.

### Compliance requirements

Participants will be required to complete a minimum of 16 of the 18 scheduled training sessions (representing >85 % attendance). Testing of outcome measures (T0, T3, T6 and T9) will be completed within 72 h of the scheduled time point, determined from the commencement of the training programme.

### Outcome measures

#### Functional strength of the lower limb

Participants will perform 1RM tests to assess maximal voluntary strength of the lower limb muscles. A standard leg press and leg extension exercise will be performed on fixed pneumatic gym equipment (Air300; Keiser). Plantarflexion will be measured bilaterally on the seated leg press by placing the toes on the footplate and using the ankle dorsiflexion to press the plate forward while maintaining 180-degree knee extension. The unweighted movement distance is marked on the equipment, allowing the researcher to ensure that full range of movement is maintained during maximal attempts. Dorsiflexion will be measured unilaterally while the participant is seated with the heel positioned on a 25-cm step. A custom-made static strap weighted with an adjustable dumb-bell will be placed over the metatarsophalangeal joint, and the participant will be required to lift the dumb-bell smoothly through 45 degrees of dorsiflexion, measured by the researcher with a goniometer. All exercises have been chosen carefully to minimise any risk of falling or injury whilst being performed.

An initial starting resistance of approximately 20 % 1RM will be estimated by the researcher to familiarise the participant with the movement, and five to ten repetitions will be performed to provide a warm-up. The researcher will then select an appropriate resistance for a near-maximal effort, and the participant will be required to perform a single repetition of the exercise while maintaining correct technique. The resistance will be increased in 5–10 % increments as appropriate, until the participant can no longer perform a full repetition. Two minutes of rest will separate each attempt, and verbal encouragement will be provided. The highest resistance used to perform a successful repetition will be recorded as the 1RM in kilograms.

#### Functional assessments

Motor function specific to PD will be assessed with section III of the UPDRS-III. The same independent assessor will perform the test at all time points and will be blinded to the intervention group. The assessment will be performed at the same time of day and will precede all other tests to ensure fatigue does not confound performance.

A Timed Up-and-Go Test (TUG) will also be performed to assess motor function and speed [29]. Participants will be asked to rise from a 46-cm chair, walk around a plastic cone located 3 m away and return to the chair as quickly as possible. The arms of the chair may be used if necessary. The test ends when the participant assumes the original seated position, and time is recorded. Regular enclosed footwear will be worn, and customary walking aids may be used if required. The average time in seconds from three trials will serve as the TUG score.

The Berg Balance Scale will be used to assess functional balance [30]. The assessment will take approximately 20 minutes and will consist of 14 static and dynamic balance tasks which are scored from 0 to 4 by a trained exercise physiologist. The same assessor will perform the test at all time points and will be blinded to the intervention group. High validity and reliability of the Berg Balance Scale in neurological patient populations have previously been established [31].

The Five Times Sit-to-Stand Test (FTSTS) will be used to assess mobility and lower limb acceleration [32]. Participants will be seated in a 46-cm chair without armrests and will be asked to begin with their arms folded across the chest and their back upright against the backrest. Participants will perform five repetitions of moving from the seated position to standing, with the legs fully extended and hip and knee joints, as quickly as possible. The total time taken to complete five repetitions will be recorded in seconds. An inertial measurement unit (Nanotrak; Catapult Innovations, Docklands, Australia) worn on the right hip, sampling accelerations and gyrations at 100 Hz, will be used to determine vertical acceleration [33]. Previous researchers have reported high validity and reliability of the FTSTS in patients with PD [34].

#### Gait

Gait will be assessed using the ProtoKinetics Zeno 4.9-metre walkway (ZenoMetrics LLC, Peekskill, NY, USA). The walkway has a 2-foot-wide grid of pressure-sensing pads with a spatial resolution of 0.5 cm and a sampling frequency of 120 Hz. Participants will be asked to perform the walking trials at their preferred walking speed while wearing comfortable footwear. Two different gait conditions will be assessed: (1) normal walking and (2) walking while counting backward in 7s from a random three-digit number, termed *dual-task gait*. Three trials of each condition will be performed in random order. A trial begins by sitting silently on a chair for 30 seconds, then standing up and walking to a starting line located 1 m from the walkway. From the start line, the participant is asked to walk up and back four times to another line 1 m beyond the end of the walkway before returning to the seated position. The 30-second rest periods between each trial allow for accurate functional near-infrared spectroscopy (fNIRS) hemodynamic brain imaging (see ‘Functional near*-*infrared spectroscopy’ section below for details). The mean asymmetry and variability will be calculated using all of the steps of all of the trials for a given condition: step velocity (in meters/second), step duration (in seconds) step length (in centimetres), double support time (in seconds) and stride width (in centimetres). Step variability will be calculated on the basis of residuals, as suggested in previous literature [35]. Gait assessments using instrumented walkways have been shown to be repeatable in older adults [35, 36].

While undertaking the gait assessments, the participants will be asked to wear an inertial measurement unit on an elastic belt (Nanotrak) for sampling of accelerations and gyrations at 100 Hz at lumbar vertebrae L3–L5, in line with the spinous processes. Harmonic ratio [37] and multi-scale sample entropy will be calculated from the recorded vertical and resultant signals [38]. These inertial measurement unit-based variables have previously been shown to be repeatable [38].

#### Posturography

Static balance will be assessed with a 45-second bipedal standing test in three separate conditions: (1) eyes open, (2) eyes closed and (3) dual-task balance (eyes open while counting backwards in serial 7s from a random three-digit number). All tests will be conducted at preferred stance width, which will be recorded at baseline and maintained during all subsequent tests. A black dot (10-cm diameter) printed on white paper will be affixed 3 m in front of the participants at eye height, and the participants will be asked to fix their gaze on the dot while undertaking the eyes-open tests. Again, 30-second rest periods between each trial will be provided for concurrent fNIRS sampling. The measurement will be conducted on a portable force plate (AccuGait; AMTI, Watertown, MA, USA) sampling ground reaction forces at 500 Hz. Instantaneous centre of pressure will be calculated on the basis of recorded signals, and centre-of-pressure travel velocity (millimetres/second) will be calculated to represent static balance. The repeatability of centre-of-pressure travel analyses has been demonstrated in previous studies [39].

#### Transcranial magnetic stimulation and electromyography

Corticospinal excitability and intra-cortical inhibition of the tibialis anterior (TA) muscle will be assessed using two Magstim 200^2^ stimulators (Magstim, Whitland, UK) connected to a 110-mm, concave, double-cone coil (maximum output 1.4 T). The coil will be positioned with anterior-to-posterior current flow, and sites near the estimated right TA motor representation (approximately 1–3 cm lateral and anterior to the vertex) will be explored to determine the optimal site, which will be marked on the scalp with a permanent marker. Resting motor threshold (RMT) and active motor threshold (AMT) will be defined as the lowest stimulator output required to achieve an MEP with an amplitude greater than 0.05 mV and 0.20 mV in six of ten stimuli, respectively. Corticospinal excitability will be determined by delivering ten single-pulse stimuli at suprathreshold intensities of 1.3 × RMT and 1.5 × RMT. In accordance with previous literature [40], SICI will be determined by delivering ten paired-pulse stimuli, with the conditioning stimulus set at 0.8 × RMT, the test stimulus set at 1.3 × RMT, and an interstimulus interval of 3 milliseconds. Intra-cortical inhibition will be determined by calculating the SICI ratio (average paired-pulse MEP amplitude/average single-pulse MEP amplitude); thus, SICI ratio values closer to 1 will represent lower levels of intra-cortical inhibition, while SICI ratio values closer to 0 will represent higher levels of intra-cortical inhibition. The total of 30 stimuli (20 single pulses and 10 paired pulses) will be delivered in sets of 5 in a randomised order.

Once the TMS protocol has been completed with the TA muscle at rest, the same protocol will be repeated in an active muscle state while the participant maintains a low-level background contraction (10 % of maximum force). The participant’s maximal voluntary isometric contraction (MVIC) of the right TA will first be determined using a force transducer (LMD500; Futek, Irvine, CA, USA) mounted in a custom-made timber frame. The participant will be seated with the knee and ankle positioned at 90 degrees and the transducer positioned above the distal end of the metatarsals. The participant will be instructed to dorsiflex at the ankle with the base of the heel remaining on the floor, producing an isometric contraction against the force transducer. The peak force obtained from three maximal efforts with 2 minutes rest between attempts with the highest force output will be recorded as the participant’s MVIC. Verbal encouragement will be provided, and visual feedback of force will be visible on a monitor directly in front of the participant at eye level. To complete the TMS protocol in the active muscle condition, 10 % of the MVIC will be calculated and indicated by a line on the monitor. The participant will be asked to maintain the force feedback level with the line while TMS is delivered, with short rest periods (5–10 seconds) between each set of five stimuli. AMT will be defined as the lowest stimulator output required to achieve an MEP with an amplitude greater than 0.2 mV in six of ten stimuli.

Maximal compound waves (M-waves) will be collected during each session and used to normalise MEP responses. This method prevents any changes in peripheral muscle excitability acting as a confounder when determining corticospinal excitability [41]. A DS7A constant current electrical stimulator (Digitimer, Welwyn Garden City, UK) will be used to deliver supramaximal stimulation to the common peroneal nerve. The head of the fibula will be palpated, and a stimulating bar will be positioned directly inferior, with light pressure applied. Stimulation will be delivered at increasing current strengths until the muscle response reaches a plateau, then increased 10–20 % to ensure maximal stimulation is achieved. The largest resulting peak-to-peak muscle response will be reported as maximal M-wave amplitude. The M-wave will be obtained in both resting and active (10 % MVIC) muscle conditions and will be used to normalise resting and active MEPs, respectively.

All muscle responses (MEPs and M-waves) will be recorded using surface electromyography (sEMG). The area of electrode placement will be shaved to remove hair, rubbed with abrasive gel to remove dead skin, and then cleaned with 70 % isopropyl alcohol. Bipolar gel Ag-AgCl electrodes (8-mm diameter, model E258S; BIOPAC Systems, Goleta, CA, USA) will be placed over the TA muscle belly with an inter-electrode distance of 2 cm. A grounding electrode will be placed over the patella and used as a common reference. The electrode sites will be marked with a permanent marker, measured in reference to the tibial tuberosity, and photographed to ensure consistent electrode placement between sessions. All cables will be fastened with tape to prevent movement artifact. All sEMG signals (including MEPs) will be amplified (×1000) with band-pass filtering between 13 Hz and 1000 Hz, digitised online at 2 KHz for 500 milliseconds, and recorded and analysed using PowerLab 4/35 (ADInstruments, Bella Vista, Australia).

#### Functional near-infrared spectroscopy

Mechanisms underlying difficulties in dual-task gait and balance are largely unclear. However, the PFC, which is primarily involved in attention and executive function, is also involved in human balance and locomotion [42], which likely plays an important role. Although attention and executive function that depend on the PFC are often affected in PD [43–45], patients may rely more on the PFC because of reduced movement automaticity of dysfunctional basal ganglia circuits [46–48]. Consequently, altered functioning of the PFC during dual-task gait and balance in PD might explain the difficulties of patients with PD and therefore will be examined in this study using fNIRS.

fNIRS neuroimaging uses optical technology to measure the relative concentrations of oxygenated haemoglobin (O_2_Hb) and deoxygenated haemoglobin (HHb) in the cortical layer microcirculation [49], producing cortical activation data consistent with data obtained from functional magnetic resonance imaging [50, 51]. Portable fNIRS has successfully been used to measure PFC activation during dual-task walking in patients with PD [52] and in other populations [53–55]. A PortaLite™ fNIRS system (Artinis Medical Systems, Elst, The Netherlands) will be used to assess PFC activation during several tasks, including counting backwards in 7s from a random three-digit number while seated and during the simple and dual-task gait-and-balance assessments described in the previous section.

The PortaLite™ device has three transmitters and one receiver, with transmitter-receiver distances of 30, 35 and 40 mm. Near-infrared light is transmitted with two wavelengths, 760 nm and 850 nm, and data will be sampled with a frequency of 10 Hz. The PortaLite™ device uses wireless technology (Bluetooth, Kirkland, WA, USA), allowing participants to perform the tasks without restriction of wires. Since a previous preliminary study showed no significant differences between left- or right-side PFC activation during dual-task walking in PD [52] the PortaLite™ device will be placed on the left forehead F3 site using the international 10–20 electroencephalography system. This location will target the left Brodmann’s areas 9 and 10, which correspond to the dorsolateral and anterior PFC [56, 57]. The device will be shielded from ambient light by covering the forehead with a black piece of fabric. Oxysoft software (Artinis Medical Systems) will be used for data collection and analysis.

On the basis of different absorption spectra, concentration changes of O_2_Hb and HHb in the targeted PFC area will be calculated from the changes in detected light intensity. This is done using the modified Beer-Lambert law, assuming constant scattering [58]. The differential path length factor, which accounts for the increased distance travelled by light due to scattering, will be set to 6 for all participants. For analysis, O_2_Hb and HHb signals of the three channels (the three transmitter-receiver distances) will be averaged. The moving SD-based movement artifact reduction algorithm method will be used within each trial to remove movement artifacts and other noise from the fNIRS signals [59]. The threshold for artifact detection will be set to 0.45 for O_2_Hb and 0.18 for HHb [52], with a window length for moving SD calculation at 0.5 seconds and a window length for artifact correction (locally weighted scatterplot smoothing window) on 1 second. The fNIRS signals will then be linearly de-trended per trial and low-pass-filtered at 0.1 Hz to remove heart rate from the signals. To enable direct comparison of the different trials within each task, the filtered signals will be biased, using the average concentration of the 5 seconds before the ‘start’ instruction as a reference (zero). Then, individual trials will be averaged per task to create three mean time course signals per person, which will then be averaged over all participants. Finally, the peak and mean concentrations (O_2_Hb and HHb) during the task and rest periods will be calculated over all trials for all participants and then averaged for each of the tasks.

### Adverse events

All adverse events will be self-reported by the participants at 3, 6 and 9 weeks and assessed by the research staff for seriousness, expectedness and causality following the guidelines recommended by the National Health and Medical Research Council (NHMRC) position statement for monitoring and reporting of safety for clinical trials (https://www.nhmrc.gov.au/guidelines-publications/e112). In this study, an adverse event will be defined as any health-related unfavourable or unintended medical occurrence (sign, symptom, syndrome, illness) that develops or worsens during the period of the trial. Adverse events will be closely monitored until resolution or stabilisation is achieved or until it has been shown that the study intervention is not the cause of the injury. Participants will be asked to contact the research staff immediately in the event of a serious adverse event. Any adverse event sustained during the exercise programme will be recorded by the trainers and immediately reported to the research staff. The chief investigator will be informed of the adverse event and shall determine its seriousness and causality in conjunction with any medical staff treating the event. A serious adverse event that is deemed related to, or suspected to be related to, the exercise intervention will be reported to the ethics committee.

### Data management and archiving

All data will be de-identified, coded and stored on a Deakin University server that can be accessed only from password-protected computers. Any physical copies of the data recording sheet will be stored in locked filing cabinets at Deakin University (Burwood). All data will be checked monthly by the chief investigator to ensure that all protocols and ethical guidelines for data collection and analysis are followed. All study-related documents will be archived at Deakin University at the end of the study for a minimum of 6 years, which is in line with current ethical requirements.

### Dissemination plan

Findings derived from the primary outcome analysis of this trial will be reported in journal articles, which will include results regardless of the direction or magnitude of the effect. The results will also be presented at leading national and international conferences and clinical forums and to other relevant health professionals and stakeholders, as well as to the participants. All investigators will have the opportunity to be listed as an author of future publications, in accordance with the Australian Government National Health and Medical Research Council guidelines [60].

### Sample size calculation and statistical analysis

Gait speed, static balance and UPDRS-III at T6 will serve as primary outcome measures, with all other assessments and time points serving as secondary outcome measures. Based on the effect size observed in a recent study using a-tDCS during walking exercise [24], it is estimated that 42 participants randomised equally to three groups will be needed. Fourteen participants in each intervention group (a-tDCS, sham, control) will provide at least 80 % power to detect a 10–15 % difference in gait speed and static balance (centre of pressure), assuming a 5 % significance level. In functional assessments, a 10–15 % difference equates to an increase of 5.2 points on the UPDRS-III, which indicates a moderate clinically important difference [61].

A two-way, repeated-measures, mixed-design analysis of variance with factors time (T0 vs. T3 vs. T6 vs. T9) and treatment (a-tDCS vs. sham vs. control) will be used to determine any effect of the intervention on balance, gait speed and measures of brain physiology. False discovery rate analysis will be applied to determine when and where significance is found. An alpha level of *P* < 0.05 will be set to determine significance.

Where possible, we will obtain endpoint measures from all withdrawals and will include all randomised subjects in the final analysis. For participants who are lost to follow-up, missing data will be handled with multiple imputation. As this approach makes an untestable assumption that data are missing at random (i.e., missing data can be predicted from the observed data) [62], we will perform sensitivity analysis to evaluate the effect of potential non-random attrition [63]. Sensitivity analyses will employ simulation and will test a range of scenarios assuming plausible arm-specific differences in outcomes for individuals who were lost to follow-up [64].

## Discussion

To our knowledge, this pilot study will be the first randomised controlled trial to combine functional PRT and a-tDCS of the M1 to target balance and gait in people with PD. The study has been designed to elucidate the functional, clinical and neurophysiological outcomes of combined PRT and a-tDCS. It is hypothesised that the combination of PRT and a-tDCS will benefit lower limb strength, postural sway, gait speed and stride variability by a greater magnitude than PRT with sham-tDCS. Further, we hypothesise that PFC activation during dual-task cognitive and gait-and-balance activities will be reduced, and that M1 corticospinal excitability and inhibition will be augmented, following the combined PRT and a-tDCS intervention.

The study is strengthened by a double-blind approach to tDCS stimulation type (a-tDCS or sham), with participants, outcome assessors and exercise supervisors remaining unaware of stimulation type for the duration of the study. Because of the nature of the exercise interventions, participants in the control group cannot be blinded; however, outcome assessors will remain unaware of allocation. The PRT programme used in this study has been developed specifically to target lower limb strength and incorporate functional balance tasks that will maximise gains in mobility and reduce postural sway. All PRT sessions will be conducted one-to-one with an experienced exercise physiologist to ensure each participant will be trained consistently. Progressions will be administered according to individual performance and will be recorded in detail to enable a post-intervention comparison, which will allow us to detect any potential inequality of exercise load between groups. Outcome measures have been carefully selected to enable us to detect functionally and clinically relevant effects of the intervention (UPDRS, TUG, FTSTS, Berg Balance Scale and 1RM lower limb strength tests), as well as to provide sensitive biomechanical analysis with previously validated techniques (step velocity, duration, length, double-support time, stride width and centre-of-pressure travel velocity). Neurophysiological assessments will provide insight into adaptive plasticity that may underpin any changes in functional capability, with TMS used to detect changes in corticospinal excitability and inhibition of the M1 lower limb representation and fNIRS neuroimaging of the PFC used during combined cognitive and motor tasks to quantify changes in PFC activation.

A limitation of this study is the reduced sample size, which has been selected for the feasibility of conducting a one-to-one, 6-week exercise intervention. Despite this, sample size calculations indicate that any clinically significant benefits of combining a-tDCS with PRT will be detected statistically. If successful, pilot data from this study are vital to informing larger-scale clinical trials in patients with PD, and the additional investigation of underpinning neurophysiological mechanisms may also be translated to inform future treatment options for a range of other movement disorders.

The safe and inexpensive nature of a-tDCS is well suited for translation to existing and established PD rehabilitation programmes and services. While current pharmacological treatments in PD provide significant benefits to reduce the impact of some motor symptoms, bradykinesia and impaired postural stability often continue to impact patients significantly [3]. These symptoms have a detrimental effect of the quality of life of patients with PD and contribute to an increased risk of falls [4]. Rates of falls among patients with PD are twice as high as those in the general older population, with 46 % of people with PD experiencing recurrent falls each year [65, 66]. Falls often result in hospitalisation [67] as well as longer-term injuries such as fractures that further restrict physical capability and contribute to a cycle of secondary health complications and a loss of independence [68]. Effective interventions targeting balance and gait will reduce the burden of PD on the individual, family and carers, as well as the healthcare system. We anticipate that the findings of this study will inform large-scale randomised controlled trials aimed at implementing PRT and a-tDCS in community- and home-based settings to treat the motor symptoms of PD.

## Trial status

Seventeen participants have completed the protocol in full, and six are currently undergoing PRT. One participant has failed to complete the intervention due to illness. Recruitment of participants is ongoing.

## Abbreviations

AMT, active motor threshold; a-tDCS, anodal transcranial direct current stimulation; fNIRS, functional near-infrared spectroscopy; FTSTS, Five Times Sit-to-Stand Test; HHb, deoxygenated haemoglobin; M1, primary motor cortex; MEP, motor evoked potential; MVIC, maximum voluntary isometric contraction; M-wave, maximal compound wave; O_2_Hb, oxygenated haemoglobin; PD, Parkinson’s disease; PFC, pre-frontal cortex; PRT, progressive resistance training; 1RM, single-repetition maximum; RMT, resting motor threshold; sEMG, surface electromyography; SICI, short-interval intra-cortical inhibition; SPIRIT, Standard Protocol Items: Recommendations for Interventional Trials; TA, tibialis anterior; tDCS, transcranial direct current stimulation; TMS, transcranial magnetic stimulation; TUG, Timed Up and Go Test; UPDRS, Unified Parkinson’s Disease Rating Scale
